# Selective Participation of Single Cortical Neurons in Neuronal Avalanches

**DOI:** 10.3389/fncir.2020.620052

**Published:** 2021-01-22

**Authors:** Timothy Bellay, Woodrow L. Shew, Shan Yu, Jessica J. Falco-Walter, Dietmar Plenz

**Affiliations:** ^1^Section on Critical Brain Dynamics, National Institute of Mental Health, National Institutes of Health, Bethesda, MD, United States; ^2^Department of Neuroscience, Brown University, Providence, RI, United States

**Keywords:** nonhuman primate, rat, prefrontal cortex, primary motor cortex, high-density microelectrode array, local field potential, whole-cell patch recording, cell assemblies

## Abstract

Neuronal avalanches are scale-invariant neuronal population activity patterns in the cortex that emerge *in vivo* in the awake state and *in vitro* during balanced excitation and inhibition. Theory and experiments suggest that avalanches indicate a state of cortex that improves numerous aspects of information processing by allowing for the transient and selective formation of local as well as system-wide spanning neuronal groups. If avalanches are indeed involved with information processing, one might expect that single neurons would participate in avalanche patterns selectively. Alternatively, all neurons could participate proportionally to their own activity in each avalanche as would be expected for a population rate code. Distinguishing these hypotheses, however, has been difficult as robust avalanche analysis requires technically challenging measures of their intricate organization in space and time at the population level, while also recording sub- or suprathreshold activity from individual neurons with high temporal resolution. Here, we identify repeated avalanches in the ongoing local field potential (LFP) measured with high-density microelectrode arrays in the cortex of awake nonhuman primates and in acute cortex slices from young and adult rats. We studied extracellular unit firing *in vivo* and intracellular responses of pyramidal neurons *in vitro*. We found that single neurons participate selectively in specific LFP-based avalanche patterns. Furthermore, we show *in vitro* that manipulating the balance of excitation and inhibition abolishes this selectivity. Our results support the view that avalanches represent the selective, scale-invariant formation of neuronal groups in line with the idea of Hebbian cell assemblies underlying cortical information processing.

## Introduction

Understanding how the collective dynamics of the cortex emerges from neuronal interactions is a fundamental challenge in neuroscience. Given the limitations in accurately recording from many neurons simultaneously, this challenge is typically approached by studying how the activity of single neurons correlates with the dynamics of the network. Of particular interest in this context has been the discovery of “neuronal avalanches” in spontaneous (Beggs and Plenz, 2003; Petermann et al., 2009; Miller et al., 2019) and evoked cortical activity (Shew et al., 2015; Yu et al., 2017) in which the collective dynamics of the cortex has been mapped using the local field potential (LFP). More specifically, it has been reliably found for slice cultures, acute slices, rodents, and nonhuman primates that the spatial and temporal spread of transient and fast deflections in the cortical LFP, when tracked using high-density microelectrode arrays (MEAs), obeys a power-law relationship in the size of LFP patterns, which is the hallmark of avalanches (Yu et al., 2014) and is in line with expectations for critical dynamics (for review see Plenz and Thiagarajan, 2007; Chialvo, 2010; Mora and Bialek, 2011; Plenz, 2012; Hesse and Gross, 2014; Marković and Gros, 2014; Muñoz, 2017).

The power law in avalanche size demonstrates that large avalanches, i.e., those that engage a large part of the cortical area monitored, are significantly more common than expected by chance (Yu et al., 2014). Hierarchical clustering *in vitro* further demonstrates that large avalanches exhibit diverse, yet distinct spatial patterns, i.e., families (Beggs and Plenz, 2004; Stewart and Plenz, 2006). This organization then raises the question of whether the activity of single neurons correlates selective with some avalanche families. Using 2-photon imaging, the spontaneous and evoked firing in groups of cortical neurons have been found to organize as scale-invariant avalanches (Bellay et al., 2015; Karimipanah et al., 2017; Bowen et al., 2019; Ribeiro et al., 2020). Similarly, extracellular unit recordings in the rodent during wakefulness, exploration, and sleep identified state-specific and repeated spike avalanche patterns (Ribeiro et al., 2016). Yet, it is currently not known how supra- and subthreshold activity of individual neurons relate to large and diverse avalanches encountered in the LFP.

Theory and experiment suggest that neuronal avalanches indicate a critical state of cortex at which numerous aspects of information processing are maximized such as dynamic range (Kinouchi and Copelli, 2006; Shew et al., 2009; Gautam et al., 2015; Shriki and Yellin, 2016; Clawson et al., 2017; Gollo, 2017) and information capacity (Shew et al., 2011; Yang et al., 2012; Fagerholm et al., 2016; Agrawal et al., 2018). Simulations suggest avalanche dynamics confer benefits as to how networks learn new input-output associations while staying adaptive (de Arcangelis et al., 2006; de Arcangelis and Herrmann, 2010; Rybarsch and Bornholdt, 2014; Stepp et al., 2015; Del Papa et al., 2017; Hernandez-Urbina and Herrmann, 2017; Michiels van Kessenich et al., 2018; Skilling et al., 2019; Zeng et al., 2019). An understanding of these beneficial aspects of avalanche dynamics concerning network properties requires insight into the cellular composition of avalanche activity.

Here, we studied the relationship between avalanche and single-neuron activity by comparing multi-site LFP recordings with simultaneously measured extra- and intracellular activity of single neurons. More specifically, when a spatial pattern of the LFP was found to repeat during a recording, we searched for reliable recruitment of single neurons during each repeated occurrence. First, we studied the extracellular unit activity and LFP signals recorded during ongoing activity from layers 2/3 of the premotor cortex in awake nonhuman primates. Since it is not feasible to separate the effects of local and distant sources of the LFP in awake animals, we next carried out complementary studies in acute slices of the rat cortex, for which the origins of the LFP signals are intrinsic to the cortex. For the slice studies, we combined intracellular whole-cell patch recordings of pyramidal neurons in layer 2/3 with multi-site LFP recordings. In line with our hypothesis, both *in vivo* and *in vitro*, we found that neurons participate selectively and reliably in particular avalanche patterns. We further demonstrate that this selective relationship between neurons and avalanches requires intact synaptic inhibition. Our findings suggest that the diversity of neuronal avalanches in the cortex emerges from diverse and distinct neuronal groups at the balance of excitation and inhibition.

## Materials and Methods

### Nonhuman Primate Recordings

**Figure 1:**
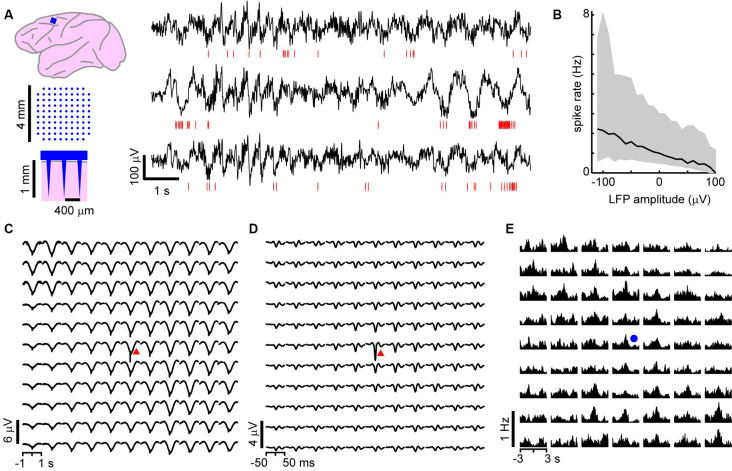
On average, local unit activity associates with negative local field potential (LFP) deflections over the spatially extended cortical area in the monkey cortex. **(A)** Left: a 96 channel electrode array (blue) was implanted within superficial layers of the premotor cortex in two macaque monkeys. Right: simultaneous recordings of awake-state ongoing LFP (black) and single-unit activity (red—spike times) from three recording sites. Units exhibit increased firing around the time of negative LFP deflections. **(B)** Unit activity increases with negative LFP amplitude. Unit spike rate as a function of LFP amplitude computed for all times (in consecutive 50 ms windows). Units and LFP recorded at the same site were compared. Displayed is the average over all sites and units for both monkeys. Large deviations from the average relationship are typical (shaded region—lower to upper quartile, line—median). **(C)** Spike-triggered average LFP waveforms indicate that widespread, slow negative LFP fluctuations are associated with local spiking (red triangle indicates the site of the local unit). LFP was band-pass filtered between 1 and 100 Hz. **(D)** Same as in panel **(C)**, but with low-frequency components of the LFP removed (band-pass, 3–100 Hz), spike-triggered average LFP waveforms indicate that local, sharp negative LFP deflections are typically associated with spiking as reported previously (Petermann et al., 2009). **(E)** The peak times of large amplitude negative LFP deflections (nLFPs; bandpass filter, 1–100 Hz) were used to compute nLFP-triggered average spike count histograms. Consistent with **(C)**, units both near to and distant from the trigger site (blue dot) exhibited significant increases in firing with no clear decaying relationship over distance. For **(C–E)** all units were compared to all LFP recordings and then averaged together keeping track of relative locations of the unit concerning the LFP recording site.

Two adult nonhuman primates (*Macaca mulatta*), one female (monkey 1; Victoria) and one male (monkey 2; Noma) were studied. High-density MEAs (96 electrodes, 10 × 10 grid configuration with no corner electrodes, 0.4 mm inter-electrode spacing, and 1.0 mm electrode length; from Blackrock Microsystems, Salt Lake City, UT, USA) were chronically implanted in the arm representation region of the left pre-motor cortex. Recordings were done at least 1 week following surgery within the context of a behavioral study during which the animals were trained to make a specific arm movement or perform a visual-motor mapping task (for details see Yu et al., 2017). Ongoing activity was recorded for 30 min during which the monkey was seated head-fixed and awake but did not perform any behavioral task. Extracellular signals were recorded at 30 kHz. In post-recording processing, LFP signals were down-sampled to 500 Hz and band-pass filtered at 1–100 Hz. One exception was the analysis presented in Figure 1D, for which the band-pass was set to 3–100 Hz.

### Spike Sorting

Extracellular signals were band-pass filtered (0.3–3 kHz) to reveal unit activity. Potential extracellular spike waveforms were detected during recording by adaptive threshold crossing (Blackrock Microsystems, Salt Lake City, UT, USA). The 400 μs preceding and 1,200 μs following each threshold crossing were stored and used for spike sorting. Manual spike sorting was performed with Plexon Offline Sorter. The first three principal components, peak-to-trough amplitude, and non-linear energy were the waveform features used for sorting. The initial waveform detection was deliberately liberal, such that it detected most unit activity as well as some noise fluctuations. The noise fluctuations provided an important baseline comparison for strict spike sorting. The degree to which a unit was different from noise was quantified with a multivariate ANOVA test (dependent variables included at least two of the waveform features). A unit was considered well isolated if the null hypothesis (unit and noise waveforms drawn from distributions with the same mean) was rejected with *p* < 0.001. If more than one unit was detected from a single electrode, each pair of units was also required to pass the same test. Moreover, each unit was required to have less than 1% of its inter-spike-intervals (ISI) less than a 1 ms refractory period.

To compute the crosscorrelation in spike count between each pair of units recorded during the ongoing activity we followed established methods (e.g., Renart et al., 2010). First, to obtain spike count vectors, the spike time stamps of each unit were: (1) binned with 1 ms temporal resolution; and (2) convolved with a Gaussian window with 50 ms width. The crosscorrelation coefficient was computed between all pairs of spike count vectors (2,145 pairs for monkey 1,780 pairs for monkey 2).

### Definition of LFP Avalanches

As established previously (Shew et al., 2009), we first detected negative LFP deflections (nLFPs) falling below a threshold of −3.5 standard deviation (SD) of ongoing fluctuations *in vivo* and −6 SD of noise *in vitro*. Unlike *in vivo*, periods of quiescence between population events were clear in the *in vitro* recordings and used to define the noise baseline. nLFPs were found to occur in clusters and their sizes were distributed according to a power law, the hallmark of neuronal avalanches. Two consecutive nLFPs (on any electrode) belonged to the same avalanche if the time interval between them was smaller than a threshold τ, which was determined using the probability distribution of inter-nLFP time intervals (Beggs and Plenz, 2003). We also repeated our analysis for different nLFP detection thresholds *in vivo*: 2.5, 3, 3.5, 4.25, and 5 SD, which has previously been demonstrated to not affect the power-law behavior in avalanche size distribution (Petermann et al., 2009). Our main findings were unchanged (see also “Definition of Avalanche Families” section). A complete scaling analysis of LFP avalanches for these two monkeys can be found in a recent publication (Miller et al., 2019).

### Definition of Avalanche Families

First, each spatiotemporal avalanche was represented as a binary spatial pattern with one bit per MEA electrode (Yu et al., 2011). Bits were set to 1 if the corresponding electrode recorded an nLFP during the event and otherwise set to 0. Next, patterns that included only one active site were excluded to minimize the potential inclusion of noise events. Then we sorted the events into families with similar binary patterns. *K*-means sorting in MATLAB (Mathworks) was employed with randomly chosen seed patterns and a Euclidean distance metric. The number *k* of families to search for was decided based on the number *N* of population events being sorted $k=\sqrt{N/\text{2}}$ (Sánchez et al., 1979).

### Family-Triggered Peri-Event Time Histograms

To characterize the relationship between every unit and every avalanche family, we computed family-triggered PETHs. If the family was comprised of *N* avalanches, then the trigger times for the PETH were the N timestamps of the first nLFPs in each avalanche. The PETHs included the 750 ms periods preceding and following the trigger times. The bins were 50 ms in width. A PETH peak was deemed “selective” if two conservative criteria were met. First, the integrated spike rate within a ±200 ms interval around the trigger time must be three times larger than the baseline spike rate computed in the two intervals −750 to −200 ms and 200 to 750 ms, relative to the trigger time. This criterion effectively reduces false positives but may classify units with very broad PETH peaks as non-selective. Second, the spike count in the ±200 ms interval around the trigger time must occur with a probability of less than 0.01 assuming Poisson spike generation of the neuron with rate λ. The rate λ was the mean spike rate calculated during the ±10 s intervals around the trigger times. The second criterion greatly reduces false positives for neurons with low firing rates, which can be common. As discussed in the main text, the number of expected false positives using these criteria was fewer than five times less than the observed number of selective unit-family pairs. To assess the delay *t* and width σ of significant PETH peaks, we fit the PETH with a four-parameter Gaussian function: *f* (*x*) = *A* + *B exp* ((− ( x − *t*)^2^) / 2 * sigma^2^) . The fit parameters *A*, *B*, *t*, and σ were determined by minimizing the summed squared differences between spike counts in each bin and the fit function. The minimization was performed with a simplex search method (MATLAB function—*fminsearch*).

### Acute Slice Preparation and Recording Media

Coronal slices from the medial prefrontal cortex (mPFC) or somatosensory cortex of Sprague–Dawley rats were cut at 400 μm thickness (VT1000S; Leica Microsystems, GmbH) in the chilled artificial cerebral spinal fluid (ACSF). In this study, we used two different types of ACSF during recordings, one for each of the two age groups. The first protocol, referred to as the *DA/NMDA* protocol in the main text, has been successfully used in prior studies (Beggs and Plenz, 2003; Stewart and Plenz, 2006) to induce avalanches in adult rats. Accordingly, slices were cut from adult rats (age 7–9 weeks) in ACSF saturated with 95% O_2_ and 5% CO_2_ (310 ± 5 mOsm) containing (in mM) 205 sucrose, 0.5 CaCl_2_, 7 MgSO_4_, 3.5 KCl, 26.2 NaHCO_3_, 0.3 NaH_2_PO_4_, 10 D-glucose. Prior to recording, slices were stored submerged at room temperature in ACSF containing (in mM) 124 NaCl, 1.2 CaCl_2_, 1 MgSO_4_, 3.5 KCl, 26.2 NaHCO_3_, 0.3 NaH_2_PO_4_, and 10 D-glucose. The recording was done in the same ACSF as used for storage, but with bath-application of 30 μM of dopamine (Sigma–Aldrich) and 5 μM of NMDA (Sigma–Aldrich). The second protocol, referred to as the *ACSF* protocol in the main text, induces neuronal avalanches in cortex slices from immature, young rats (Shew et al., 2010) in which we recorded under normal ACSF perfusion, but stored slices in a modified ACSF before recording. In the modified storage ACSF, Na was replaced with choline and spontaneous population activity arises without further pharmacological manipulation when switching to the recording ACSF. Moreover, this protocol may provide a practical *in vitro* model for the study of cortical regions that have reduced dopamine receptor density. For this protocol, slices were cut from young rats (ages 2–3 weeks) in modified ACSF containing (in mM) 124 choline-Cl (Sigma–Aldrich), 1.2 CaCl_2_, 1 MgSO_4_, 3.5 KCl, 0.3 NaH_2_PO_4_, 26.2 NaHCO_3_, 10 D-glucose, and saturated with 95% O_2_ and 5% CO_2_ (310 ± 5 mOsm). Note that choline replaces the sodium of normal ACSF. The slices were stored submerged at room temperature in the same modified ACSF as used for slicing. MEA recordings were performed in normal ACSF.

All recordings were performed with ACSF saturated with 95% O_2_ and 5% CO_2_, perfused at 3–4 ml/min at 35.5 ± 0.5°C. Disinhibited activity was recorded by bath-application of picrotoxin (50 μM, Sigma–Aldrich) to the respective normal recording medium.

### Recording LFP Avalanches *In vitro*

Spontaneous LFP activity was recorded with integrated planar MEAs (Multichannel Systems; GmbH) that contained 59 electrodes arranged on an 8 × 8 grid with an inter-electrode spacing of 200 and 30 μm electrode diameter (four corner electrodes and one ground electrode missing). Extracellular signals were recorded with a 1 kHz sample rate and low-pass filtered between 1 and 200 Hz to obtain the LFP. The activity was recorded for 20–45 min. Experiments with fewer than 100 nLFPs were not included in our analysis. Avalanches and avalanche families were defined as described above for the *in vivo* recordings.

### Whole-Cell Patch Recordings

The intracellular patch solution contained (in mM) 132 K-gluconate, 6 KCl, 8 NaCl, 10 HEPES, 0.2 EGTA, 2.2 Mg-ATP, 0.39 Na-GTP (Sigma–Aldrich). The pH was adjusted to 7.2–7.4 with KOH. The final osmolarity of the pipette solution was 290 ± 10 mOsm. Biocytin hydrochloride (0.3%; Sigma–Aldrich) was added to the pipette for use in post-fixation (4% paraformaldehyde) anatomical reconstruction. A putative pyramidal cell (~100 μm from the slice surface) was visually identified by its somatic shape and prominent apical dendrite and later confirmed by reconstructed morphology and/or electrophysiology. Intracellular membrane potentials were recorded in current-clamp mode (Axopatch 200B, Axon Instruments, Missouri City, TX, USA), pre-amplified and low-pass filtered at 10 kHz (Cyberamp380, Axon Instruments, Missouri City, TX, USA), and digitized at 25 kHz for voltage and 5 kHz for current using the CED 1401 (Cambridge Electronic Design, UK). Data were collected with Spike2 (CED) and analyzed off-line. Neurons were included in the analysis if their membrane potential was stable below −60 mV and if their action potential half-width was <2.5 ms (see Table 2 for the presentation of more electrophysiological parameters). To visualize the morphology of patched cells (e.g., Figure 5B), a subset of slices (*n* = 9) was post-processed with streptavidin-conjugated Texas Red (Molecular Probes, Inc.), imaged with a Zeiss LSM 510 confocal microscope, and were stitched, projected and traced offline using Fiji ImageJ^1^.

**Table 1:** Comparison of *in vitro* population activity for the three different recording conditions (mean ± SEM).

	DA/NMDA	ACSF	PTX
Number of slices (*n*)	85	42	9
Spontaneous activity duration (s)	1,486 ± 538	1,586 ± 667	1,306 ± 629
Total nLFP count from all sites (*n*)	596 ± 810	5,700 ± 9,130	4,733 ± 6,677
Rate of nLFPs at single site (Hz)	0.38 ± 0.46	3.7 ± 4.8	4.3 ± 5.1
Integrated nLFP amplitudes from all sites (mV)	−5.8 ± 7.1	−80 ± 113*	−158 ± 186
Number of families/ experiment *(n)*	14.9 ± 2.4	17.61 ± 5.4	6.6 ± 3.1*
Families w/neuron response (%)	6.7 ± 2.9	5.7 ± 2.9	42.9 ± 34.9*

*p < 0.05.

**Table 2:** Action potential electrophysiological parameters for whole-cell patch recordings of pyramidal neurons (mean ± SEM).

	DA/NMDA	ACSF	PTX
Number of cells *(n)*	71	36	12
Resting potential (mV)	−71 ± 4	−68 ± 5	−71 ± 5
Action potential threshold (mV)	−35 ± 5	−38 ± 4	−34 ± 6
Action potential amplitude (mV)	88 ± 9	87 ± 9	87 ± 7
Action potential width (ms)	1.0 ± 0.4	1.68 ± 0.6*	1.0 ± 0.5
After hyper-polarization	8.8 ± 2.7	8.2 ± 2.5	10 ± 4
amplitude (mV)
After hyper-polarization time (ms)	19 ± 7	21 ± 6	16 ± 11

*p < 0.05. Only neurons for which reliable action potential measures were obtained are listed.

**Figure 2:**
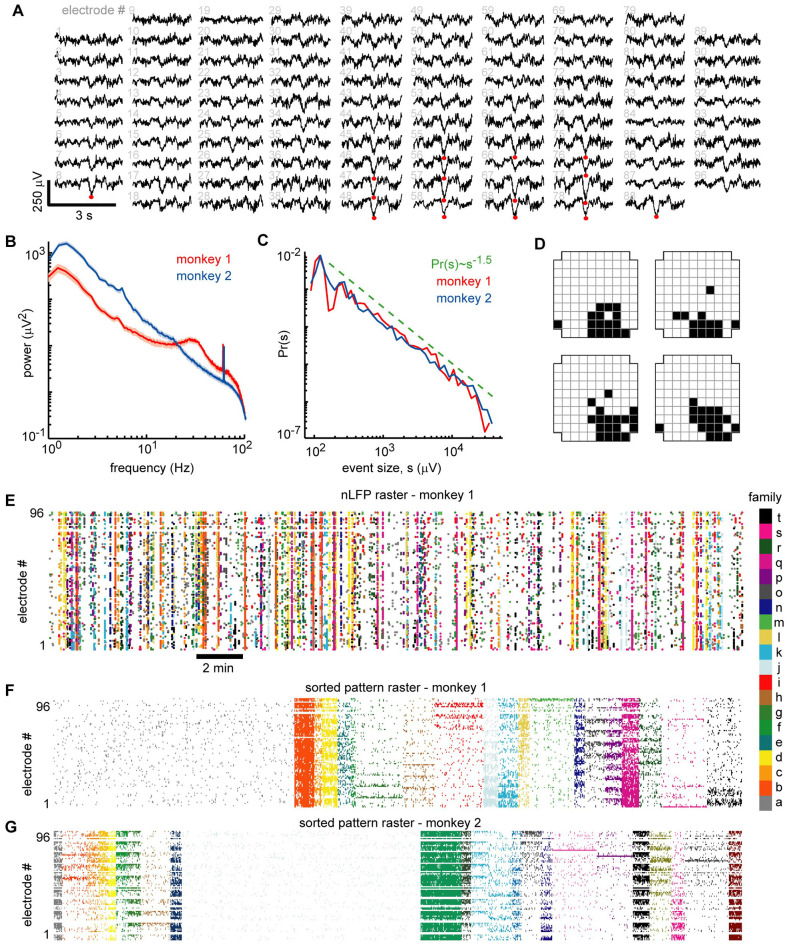
Ongoing neuronal avalanches are composed of repeating spatial nLFP patterns. **(A)** Displayed are 3 s LFP recordings arranged to match the spatial layout of the 96 recordings sites. Multiple nLFPs (red dots) often occurred together within ~100 ms across multiple sites—we defined such occurrences as “population events” (see Experimental Procedures). **(B)** Power spectra of LFP were broadband showing that low-frequency fluctuations dominate the signal. A prominent ~30 Hz oscillation was present in monkey 1 (see also Miller et al., 2019). All recording sites were analyzed—median (line) and lower to upper quartile (shaded region) are shown. **(C)** Distributions of population event sizes *s* demonstrate that the activity is neuronal avalanches, defined by *Pr(s) ~ s^−1.5^* (Beggs and Plenz, 2003; Petermann et al., 2009). **(D)** The spatial locations of nLFP avalanches were represented with a binary pattern (Yu et al., 2011). The upper left pattern corresponds to population event in panel **(A)**. The other three patterns were similar but occurred at different times and are shown as typical like-examples extracted by our algorithm use. **(E)** Raster of all nLFP times and locations. Vertical clusters of nLFPs with matching color belong to one avalanche. **(F)** Avalanches were sorted into families with like patterns. The sorted avalanche raster of monkey 1 is shown. Color code identifies family and is same as in panel **(E)**. Note that family *a* (gray, left) is comprised of typically small avalanches that were not similar to many others. **(G)** Sorted avalanche raster of monkey 2.

**Figure 3:**
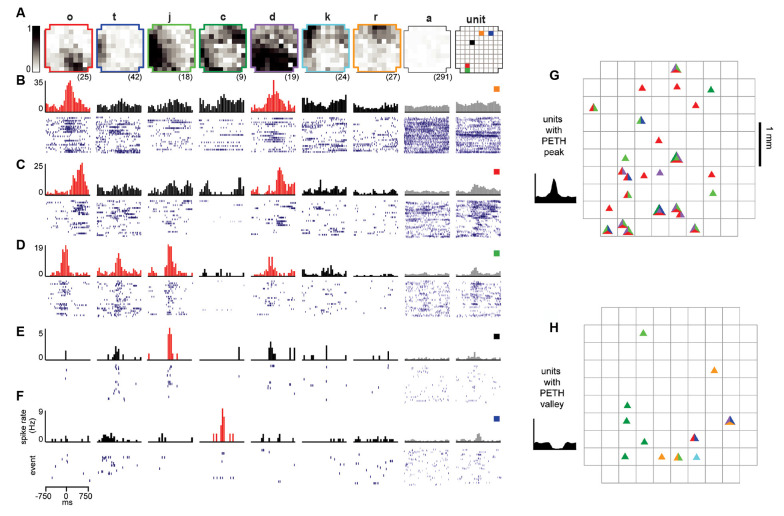
Reliable ensembles of spiking units underlie avalanche families. **(A)** The average pattern for eight example avalanche families. Letter family labels correspond to Figures 2E,F of monkey 1. Grayscale indicates the fraction of events in which the site participated. Right: color code for locations of units in panels **(B–F)**. **(B–F)** Each column displays family-triggered spike rate PETHs (top) and spike rasters (bottom) for five example units. For example, the left-most PETH and spike raster in **(B)** indicates that this unit increases its firing rate selectively during the occurrence of family *o*. The temporal bin width for the PETHs was 50 ms. The second to right column shows the average response to avalanches of size 1 (pattern a), excluding large avalanche families. The right-most column includes PETHs and rasters triggered on all nLFPs recorded at the same site as the unit, disregarding family categorization. Red histograms indicate the family specificity of the unit. **(G,H)** Array location of units (triangles) that selectively increased **(G)** or decreases **(H)** their firing in response to families shown in panel **(A)**. The colors within each triangle indicate which families the unit was selective for.

**Figure 4:**
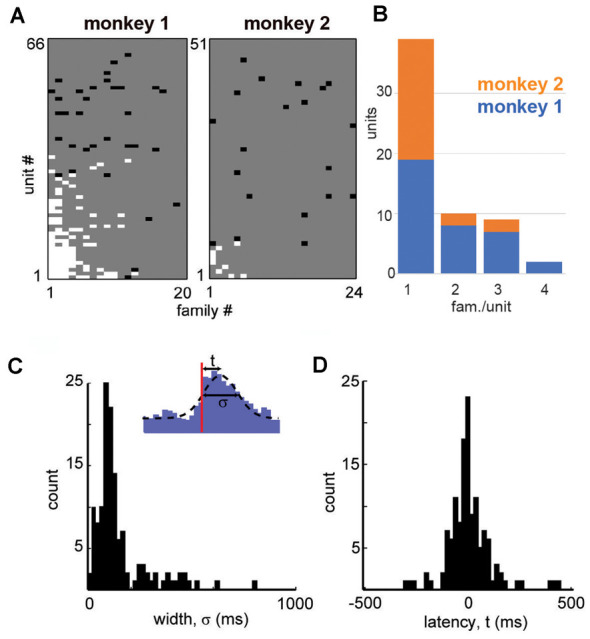
Summary in family selectivity and temporal precision in a unit-family relationship. **(A)** Summary of all unit-family relationships for both monkeys. White pixels indicate firing increase, black pixels indicate a decrease in the firing. **(B)** Histogram of units and the number of selective families for both monkeys. **(C)** We estimated the latency *t* to peak firing and the width σ of the PETH peak for each strong unit-family pair, by fitting a Gaussian function with an offset (*inset*). The distribution in PETH widths peaked around 100 ms but was also widespread. **(D)** Peak unit firing centered near *t* = 0 but could precede or follow the occurrence of a family by up to 100 ms.

**Figure 5:**
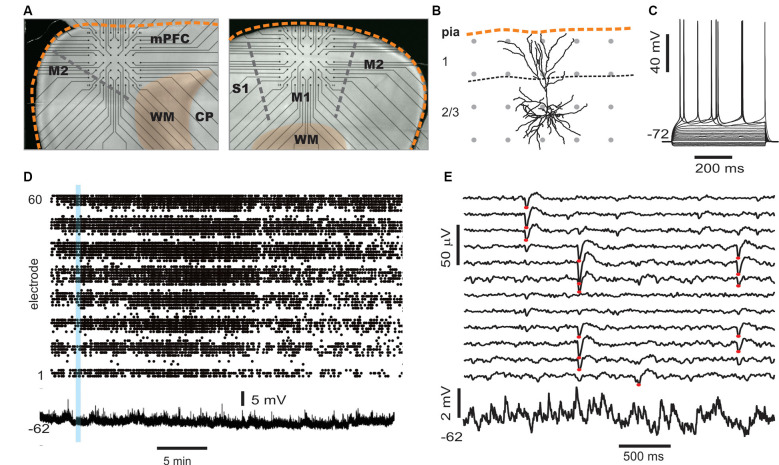
Simultaneous multi-site LFP and whole-cell patch recordings *in vitro*. **(A)** Trans-illuminated pictures that display placement of acute coronal slices from rat cortex on a planar microelectrode array (MEA), visible as straight connection leads ending in recording electrodes (*black dots*). Left: example for medial prefrontal cortex (mPFC) recordings. The medial cortex border is oriented upwards. Right: example of somatosensory cortex (M1) recording. The dorsolateral axis is oriented upwards. Scale: inter-electrode distance is 200 μm. *Orange broken line*: cortical border. WM, white matter; CP, caudate-putamen. **(B)** Example of a reconstructed layer 2/3 pyramidal neuro (confocal image) to illuminate size relationships between single neurons and spacing of MEA electrodes (*gray dots*). **(C)** Sub- and suprathreshold voltage responses to step current injections of a whole-cell patched pyramidal cell. **(D)** Example of spontaneous nLFP activity on the full array (electrodes 1–60 ordered in groups of eight per row) in the presence of NMDA/DA, which typically lasts for >30 min (Stewart and Plenz, 2006). The time course of the simultaneously recorded intracellular membrane potential from a whole-cell patched neuron is displayed at the bottom. **(E)** Five seconds of ongoing LFP population activity recorded from a subset of MEA electrodes spanning layer 2/3 and intracellular membrane potential taken from the period in panel (**D**; *blue bar*) at higher spatiotemporal resolution. *Red dots* indicate suprathreshold nLFPs. Note the absence of any apparent, straightforward relationship between single-neuron activity and nLFPs.

## Results

### Extracellular Units and LFP During Ongoing Activity in Awake Nonhuman Primates

We first studied the relationship between LFP-based avalanches and single-neuron activity in the ongoing activity of nonhuman primates. LFP recordings (1–100 Hz) were performed with high-density MEAs chronically implanted towards superficial layers of the premotor cortex over the arm representation region in two macaque monkeys (Figure 1A). During the 30 min recordings, the monkeys were awake, but not engaged in a task. Spike sorting was used to identify 66 and 51 well-isolated extracellular units in monkeys 1 and 2, respectively (for details see “Materials and Methods” section). The firing rates of the units were 3.6 ± 9.4 Hz (mean ± SD) ranging from 0.03 to 52 Hz. The trough-to-peak time difference of unit waveforms was 345 ± 140 μs. We found average pairwise spike correlation coefficients of 0.050 ± 0.002 and 0.015 ± 0.001 for our monkeys 1 and 2, respectively consistent with previous reports (e.g., Ecker et al., 2010; Renart et al., 2010). In line with previous studies (Gray and Singer, 1989; Murthy and Fetz, 1996; Destexhe et al., 1999; Pesaran et al., 2002; Nauhaus et al., 2009; Petermann et al., 2009; Kelly et al., 2010; Okun et al., 2010), we observed a tendency for units to coincide with negative excursions in the LFP (Figure 1A). This was quantified by computing the spike rate as a function of LFP amplitude recorded within a 50 ms windows at the same site. In Figure 1B, which displays the average over all units and all times, we show that the rate increases with negative LFP amplitude as reported previously for high-density arrays based on tungsten electrodes (Petermann et al., 2009).

### Averages Reveal Non-selective, Widely Distributed Unit-LFP Relationships *In vivo*

Having demonstrated that the LFP and extracellular units are related at individual electrodes, we next explored traditional spike-triggered and LFP-triggered relationships for our recordings to identify spatial selectivity in the LFP or unit activity concerning activity on the array. The example in Figures 1C,D, in which the location of the trigger unit is marked by the red triangle, draws attention to the spatially widespread, seemingly non-selective average nLFP activity related to local spiking. When slow LFP fluctuations were included in the analysis, i.e., band-pass filtering between 1 and 100 Hz, we found that the spike-triggered LFP waveform exhibited a broad (~0.5 s negative deflection with a minimum close to the trigger time, Figure 1C). When very slow fluctuations were excluded, by band-pass filtering the LFP between 3 and 100 Hz, the spike-triggered average LFP waveform displayed a sharp (~20 ms) negative peak with the largest amplitude at the recording site nearest the triggering unit, in line with previous studies (Nauhaus et al., 2009; Petermann et al., 2009), yet it systematically decayed with distance from the recorded unit (Figure 1D). This suggests a rather non-selective spatial relationship between low-frequency components in the LFP and single-unit activity. Next, we computed LFP-triggered averages of unit activity using the peak times of the nLFPs for triggers (Figure 1E). We considered all nLFPs that fell below −3.5 SD. For this and the remainder of the analysis in this article, we studied the 1–100 Hz frequency band of LFP signals. Consistent with previous studies in awake animals (Destexhe et al., 1999; Petermann et al., 2009), we found that peri-event time histograms (PETHs) of unit counts often indicated peak firing centered on the nLFP times. Consistent with Figure 1C, units that were distant from the nLFP recording site displayed a PETH peak that was comparable with that of nearby units, on average.

Figure 1 demonstrates that, on average, the spiking activity of single neurons is related to the LFP signal. However, the spike-triggered average LFP waveform for the average unit peaks around 1–10 μV (Figures 1C,D), which is much smaller than the 100 s of μV moment-to-moment fluctuations in the LFP (see Figure 1A; standard deviation over all electrodes was 35 ± 5 μV). Similarly, the nLFP-triggered spike histogram revealed an average increase in firing of less than 1 Hz (Figure 1E), which is a small change relative to ongoing 100 s of Hz fluctuations in spike rate. The coefficient of variation for the ISI distributions was 2.2 ± 0.5 and the standard deviation of instantaneous spike rates (1/ISI) was 75 ± 35 Hz.

### Moment-to-Moment Fluctuations in the Spatial LFP Are Organized as Avalanche Families

These observations raise the question to what extent do average relationships faithfully represent the moment-to-moment relationships between spiking and LFP signals? The analysis that follows was designed to answer these questions and consisted of three main steps. First, we identified neuronal population events based on the spatial patterns of LFP signals afforded by multi-site recordings. Second, we sorted the population events into “families” of like events, based on which sites exhibited negative LFP deflections during each event. Third, we tested each unit individually for family-specific changes in the firing. If our hypothesis is correct, we should find that certain units fire selectively during certain families, while other units prefer other families.

Our definition of a population event is motivated by two observations: (1) nLFPs are associated with increased spiking activity; and (2) LFP signals recorded simultaneously from different sites are often highly correlated (e.g., Figures 1A, Figure 2A; Destexhe et al., 1999; Leopold and Logothetis, 2003; Nauhaus et al., 2009). Therefore, we define a population event to be a set of nLFPs (typically from many recording sites), which occur together sufficiently close in time. Specifically, if the time interval between two consecutive nLFPs is less than a threshold τ, we assign them to the same population event. The threshold τ is chosen based on the inter-nLFP-interval distribution, which was bimodal; τ is between the peaks in the distribution, thus distinguishing the long time-scale which separates events and the short time-scale of within-event nLFPs (τ = 130, 114 ms for monkey 1, 2). In line with previous studies of ongoing activity in nonhuman primates (Leopold and Logothetis, 2003; Miller et al., 2019), slow timescale dynamics were dominant, although monkey 1 did show a slight increase in gamma-band power near 30 Hz compared to the 10–20 Hz range (Figure 2B). We recorded 1,308 and 2,016 population events for monkeys 1 and 2, respectively. Population events were diverse in spatial extent, spanning 9.6 ± 16.5 and 9.4 ± 16.2 electrodes (mean ± SD) for monkeys 1 and 2. We defined the size of a population event as the summed amplitudes of all the nLFPs comprising the event and demonstrate that the distribution of the population event sizes was close to a power-law with exponent −1.5 (Figure 2C). This power-law event size distribution indicates that the dynamics we study here are “neuronal avalanches” (Beggs and Plenz, 2003), in line with previous studies of ongoing activity in the cortex of awake monkeys (Petermann et al., 2009; Klaus et al., 2011; Miller et al., 2019). Next, for each avalanche, we generated a representative binary 10 × 10 pixel pattern (corner electrodes missing), which indicates which sites were active during the event (1 = active, 0 = inactive; Yu et al., 2011). Figure 2A exemplifies 3 s of simultaneous LFP recordings with an avalanche occurring about 1.5 s into the example (red dots mark nLFPs). The upper left in Figure 2D shows the corresponding binary pattern for this occurrence. We then used a *k*-means algorithm to find families of avalanches with similar activation patterns (Beggs and Plenz, 2004; Stewart and Plenz, 2006). Four example patterns from one family are shown in Figure 2D. The nLFP raster in Figure 2E shows all nLFP times and sites during a 30 min recording from monkey 1 with corresponding color-coded avalanche families. The occurrence-times of events in one family were typically scattered throughout the 30 min recording. Figure 2F displays the corresponding binary patterns derived from the nLFP raster sorted into families of similar patterns. The sorted raster of binary patterns for monkey 2 is shown in Figure 2G.

We note that *k*-means sorting resulted in spatially wide-spread patterns. It also results in one “misfit” family comprised of many small and local events that repeated rarely during the recording and will not be considered further for this analysis (e.g., family *a* in Figure 2F). Our objectives and the results of the *k-means* sorting were only to establish several families, within which events had similar spatial patterns of activation. Practically, as *k* is reduced, families include more population events and units become less selective for families. We quantified this trend by computing the ratio of the peri-event time histogram (PETH) peak height *H_f_* of selective unit-family pairs to the nLFP-triggered PETH peak height *H_nf_*, which disregards families. We found that for *k* = 30, 20, 10, 5, 4, 3, the ratio *H_f_*/*H_nf_* = 4.8, 4.4, 3.4, 3.2, 2.2, 1.0, respectively (monkey 1). Note that $\sqrt{N/\text{2}}$ = 20 for monkey 1. Our main conclusions are not qualitatively affected by changes in *k* between 10 and 30. Higher values of *k* tended to reduce the number of events in each family, resulting in poor statistics. Moreover, using hierarchical clustering as reported previously (Beggs and Plenz, 2004; Stewart and Plenz, 2006) did not significantly change our results (data not shown). We also repeated this analysis for different nLFP thresholds and found that for thresholds of 2.5, 3, 3.5, 4.25, and 5 SD, the ratio *H_f_*/*H_nf_* = 2.6, 3.5, 4.4, 6.0, 4.7, respectively (monkey 1). Thus, for all thresholds, we found a greater than 100% increase in selectivity compared to nLFP-triggered PETHs which disregard families of population events.

### Unit-Firing Is Selective for Avalanche Families

With the population events sorted into avalanche families, the next step was to determine whether units fired selectively for families. To accomplish this, we computed the family-triggered spike rate PETHs. One PETH was computed for each unit-family pair, triggered on the times of the first nLFP in each family. Examples for five units and a subset of families are displayed for monkey 1 in Figures 3A–F. The large spatial extent of each family is clearly visible in the family averages (Figure 3A) contrasted by the selectivity in family-triggered PETHs for units (Figures 3B–F; red histograms). Our main finding was that extracellular units were reliably and selectively active for avalanche families identified in the LFP. Some units were reliably active during multiple families (e.g., families *o* and *d* in Figures 3B–D), while other units (Figures 3E,F) fired reliably for only one family. The locations of the five units which fired reliably for families in Figure 3A were distributed diffusely over the majority of the 4 × 4 mm recording region (Figure 3A; right). The locations of all units in monkey 1 that were selectivity for the families displayed in Figure 3A are shown in Figures 3G,H for firing increase and decrease, respectively.

A closer inspection of the color-coded locations of the units reveals that spatial location is not predictive for family selectivity. For example, unit orange did not respond to family *r* who overlapped with its location but was selective for family *o* and *d*, who are most active at non-overlapping locations on the array.

All units and their respective family selectivity with corresponding negative or positive modulation are summarized in Figure 4A for both monkeys. In monkey 1, we found 124 selective unit-family pairs with a strong change in firing revealed by the family-triggered PETH. In monkey 2, we found 29 selective unit-family pairs. Here, we adopt a conservative definition of “selective,” requiring a strong increase or decrease in firing compared to baseline. In both monkeys, the number of strong relationships was more than five times greater than the number expected by chance (nine and five for monkeys 1 and 2), demonstrated by repeating our analysis with randomized spike times—each time was shifted by a random amount between 1 and 10 s. Most units were selective for only one family as shown in the distribution of unit selectivity for families for both monkeys in Figure 4B. Units selective for multiple families could exhibit various combinations in the direction of modulation. Several units were positively modulated by some families but negatively modulated by other families (Figure 4A; units with at least 1 white and one black square in a row, M1 #1, 3, 17, 20, 28, 29 and M2 #8). On the other hand, some units were only negatively modulated by multiple families (Figure 4A; units with >1 black square in a row, M1 #35, 36 and M2 #19).

Among the units and families that were strongly related, we found that the temporal precision of unit participation in families was varied. For example, Figures 3B,C reveal PETH peaks that are broader than those in Figures 3E,F. Moreover, the latency from trigger time to PETH peak also varied. To quantify the width and latency of the PETH peaks, we fit a Gaussian function to the PETH (Figure 4C, inset, Experimental Procedures). We found that the PETH peak widths, i.e., standard deviation parameter of the Gaussian fit, were 140 ± 136 ms and 170 ± 110 ms and the latencies were broadly distributed 5 ± 107 ms (mean ± SD) and 0.4 ± 106 ms for monkeys 1 and 2 respectively (Figures 4C,D). Both width and latency variability suggest the temporal precision of unit-family relationships to be of the order of 10–100 ms.

The grand average nLFP-triggered spike histograms and spike-triggered average LFP shown in Figure 1 conceals the richness of the relationship between different units and different families of LFP population events. For example, comparing the nLFP-triggered spike histograms in Figure 1E to the family-triggered PETHs in Figure 3, we see that family-triggered PETHS often had much larger or sharper peaks. This can also be seen by comparing the family-triggered PETHs to the rightmost PETHs in Figure 3, which were triggered on the times of all nLFPs that occurred on the electrode which recorded the unit. Quantitatively, we found that the selective unit-family pairs (as defined above) exhibited a PETH peak that was 4.4 ± 8.8 times larger than the nLFP-triggered PETH peak for monkey 1 and 4.3 ± 6.9 times larger for monkey 2. These results demonstrate that if all units and population events are averaged together as in Figures 1B–E, one underestimates the strength and spatiotemporal complexity of the relationship between unit activity and the LFP.

### Synaptic Inputs to Layer 2/3 Pyramidal Neurons Selectively Occur During Avalanche Families in Rat Acute Slices

We have shown above that select ensemble of spiking neurons are closely related to LFP-based avalanche patterns in the cortex of awake monkeys. We next carried out combined whole-cell patch-clamp and multi-site LFP recordings in acute slices of rat somatosensory and medial prefrontal cortex (Figure 5). Since afferent fibers from distant regions are severed in the acute slice, this preparation allows us to investigate the selectivity of intrinsic dynamics in local cortical circuits. We focused on the role of layer 2/3 pyramidal neurons and carried out control experiments with pharmacologically blocked fast GABA_A_-receptor-mediated synaptic inhibition.

In a first set of *in vitro* experiments, population activity was elicited in acute coronal slices from medial prefrontal cortex (mPFC) and motor cortex (M1) of adult rats (age 7–9 weeks) induced by continuous bath application of 30 μM dopamine (DA) and 3 μM NMDA in ASCF as reported previously (Beggs and Plenz, 2003; Stewart and Plenz, 2006). In the second set of experiments, slices were taken from mPFC and M1 of young rats (age 2–3 weeks) using a choline-based, protective slicing solution followed by recording spontaneous activity in normal ACSF only (for details see Experimental Procedures). Multi-site LFP was recorded using planar 60-electrode MEAs covering a 1.6 × 1.6 mm^2^ region with an interelectrode distance of 200 μm (Figure 5A). There were several notable differences in basic parameters between these two protocols. Spontaneous LFP activity in normal ACSF of young slices was about 10 times higher in rate and aggregate LFP amplitude compared to the DA/NMDA induction protocol for slices from adult rats (Table 1). We identified all cells as putative pyramidal neurons based on a combination of morphology, I/V-responses, and action potential properties (Figure 5B; Table 2). For most cells, we also obtained extensive measures of action potential firing, which demonstrated that the increase in LFP activity for the younger slices correlated with a significantly longer action potential width for pyramidal neurons typical for immature neurons (Table 2). Thus, the two protocols allowed for examining avalanche and single-neuron activity under two largely different rates of activity. Except where noted, the following observations were found for both protocols. We defined neuronal avalanches as described previously (Beggs and Plenz, 2003; Stewart and Plenz, 2006) and sorted them into families exactly as in the *in vivo* data analysis.

An example of a simultaneously recorded intracellular membrane potential from a layer 2/3 pyramidal neurons and the spontaneous LFP on the MEA is shown in Figures 5B,C. Upon wash-in of DA/NMDA, ongoing LFP activity emerged and the intracellular membrane potential depolarized by ~4.0 ± 3.5 mV (Figures 5D,E).

**Figure 6:**
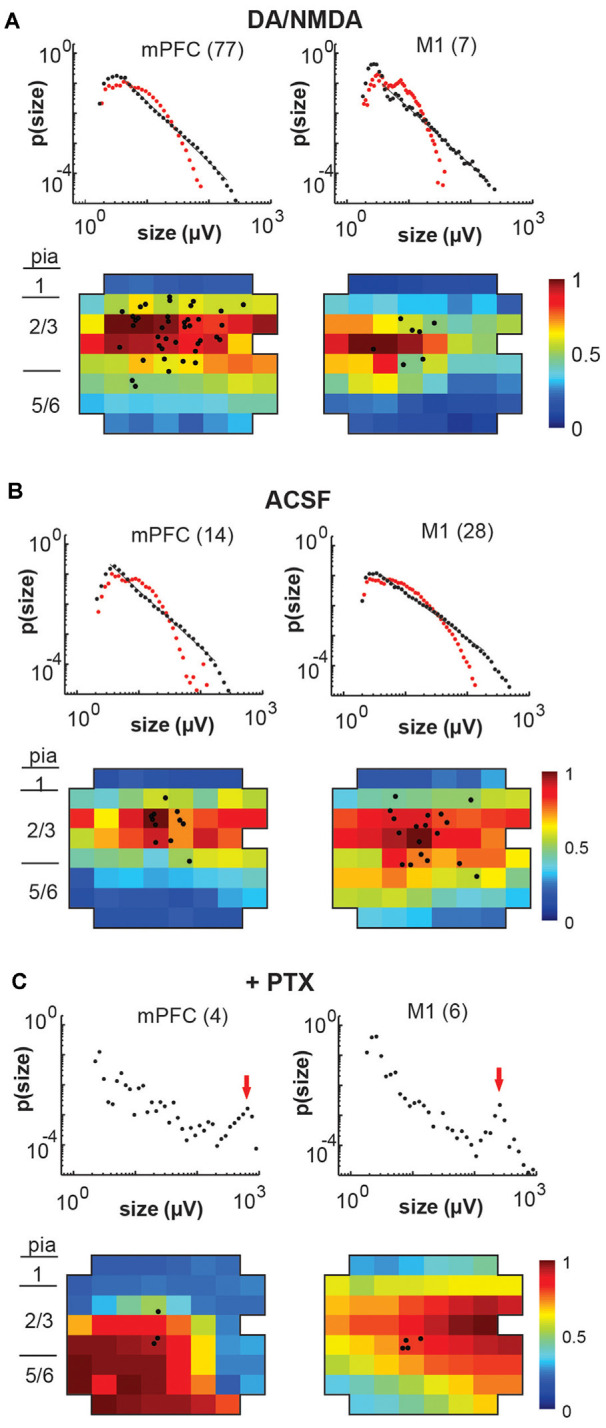
Overview of total avalanche activity and location of whole-cell patched pyramidal neurons in layer 2/3 for all recording conditions. **(A)** Avalanche size distribution obtained under artificial cerebral spinal fluid (ACSF) + DA/NMDA conditions for mPFC and M1 acute slices from adult rats. Size distributions reveal power laws in line with neuronal avalanches, which are destroyed by shuffling (*red*). Numbers indicate the total number of slice experiments for each region. Bottom density plots indicate population activity measured on the MEA aligned to medial (mPFC) and dorsal (M1) border averaged over all slice experiments. Approximate layers as a visual guide indicated on the left (MEA, 8 × 8 electrodes, 200 μm interelectrode distance, corner electrodes missing, additional ground electrode in 4th row on the right). The color indicates the activation rate (averaged over experiments). Black markers indicate soma locations of patched neurons on the array. **(B)** Same as in panel **(A)** for ACSF condition in slices from young rats prepared under-protected choline condition. **(C)** Disinhibited condition due to the addition of picrotoxin (+PTX) for slices from adult and young rats combined. Red arrows point to the predominance of system size spontaneous activations.

We note that, unlike our *in vivo* recordings in which the MEA matrix was placed horizontally within layer 2/3, the *in vitro* MEA spanned multiple cortical layers across the coronal slice with the uppermost row placed along the medial (mPFC) or dorsal/dorsolateral border of the cortex (M1). In line with our previous reports (Stewart and Plenz, 2006, 2007; Petermann et al., 2009), we found that LFP activity occurred predominantly in layer 2/3 (Figure 6A) for both protocols. In line with our *in vivo* observations, these predominantly layer 2/3 nLFP patterns distributed in sizes according to a power law that was sensitive to temporal shuffling, again as shown in our original article on neuronal avalanches in the acute cortex slice (Beggs and Plenz, 2003; Figures 6A,B).

**Figure 7:**
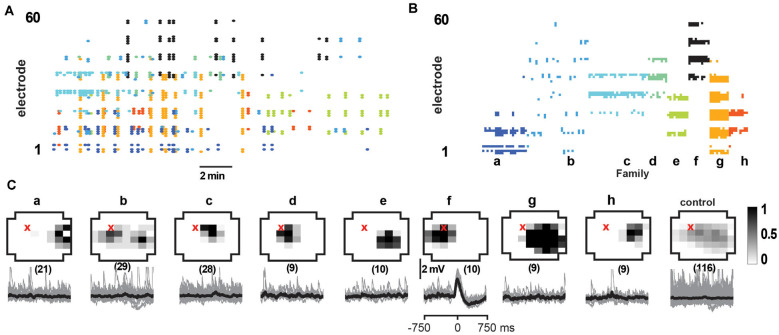
Select responses in layer 2/3 pyramidal neurons to families of NMDA/DA induced LFP avalanches. **(A)** nLFP raster on the MEA with color-coded avalanche families and their temporal occurrences during spontaneous activity (15 min recording, mPFC). **(B)** Corresponding family-sorted avalanche raster. **(C)** Top: average spatial LFP pattern for all avalanche families. Grayscale indicates the fraction of site participation for each family. The red cross marks the soma location of the patched pyramidal neuron. *Control* includes all avalanches except those in family *f*. Bottom: family-triggered average intracellular membrane potential time course (*black*). *The number in brackets* indicates family members. Pre-averaged individual traces are shown in *gray*. Family *f* generated reliable input to the patched cell. Corresponding *control* demonstrates no reliable trigger with other family patterns outside *f*.

As observed *in vivo*, LFP avalanche patterns were very diverse, but certain patterns tended to repeat during a recording. Figure 7A displays an unsorted nLFP raster of avalanches indicating the color-coded families and their time of occurrence. Figure 7B shows the corresponding sorted raster into avalanche families. Since action potential firing was very low in the patched neurons (<1 Hz), our goal here was to test whether neurons displayed significant subthreshold membrane potential changes concerning particular families. To this end, we performed family-triggered averages of the membrane potential recordings.

**Figure 8:**
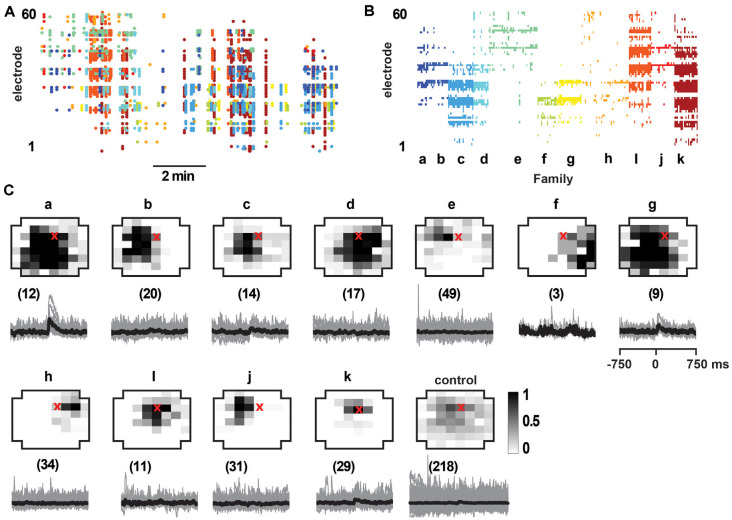
Select responses in layer 2/3 pyramidal neurons to avalanche families in normal ACSF from young slices. **(A)** nLFP raster on the MEA with color-coded avalanche families and their temporal occurrences during spontaneous activity in young slices (10 min recording, M1). Note higher rate of avalanche occurrence compared to slices from adult rats (see Figure 7). **(B)** Corresponding family-sorted avalanche raster. **(C)** Top: average spatial LFP pattern for all avalanche families. Grayscale indicates the fraction of site participation for each family. The red cross marks the soma location of the patched pyramidal neuron. *Control* includes all avalanches except those in families *a, g*. Bottom: family-triggered average intracellular membrane potential time course (*black*). The number in brackets indicates family members. Pre-averaged individual traces are shown in *gray*. Note that families *d* and *i* do not reveal significant intracellular responses despite substantial spatial overlap with soma location. Families *a* and *g* correlated with reliable intracellular responses in the patched pyramidal neuron. Corresponding *control* demonstrates no reliable trigger with non-*a*, *g* families.

Our main finding from the *in vitro* recordings was that pyramidal neurons displayed reliable subthreshold membrane potential responses only from select avalanche families, in line with our selectivity results *in vivo*. Examples of family-triggered changes in membrane potentials for one neuron using the DA/NMDA protocol are shown in Figure 7 below the average activity pattern for the corresponding families in Figure 7C. A corresponding example from the normal ACSF protocol for young slices is shown in Figure 8.

**Figure 9:**
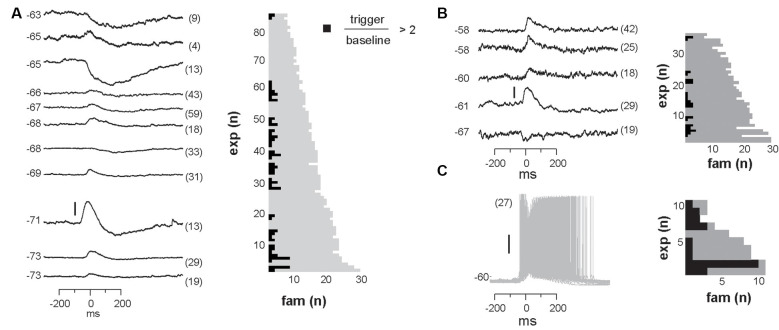
Summary of significant membrane potential responses within each experimental group. **(A)** NMDA/DA condition in slices from adult rats: 42 neurons were found to receive reliable and selective input from particular avalanche families. Left: the subset of 11 family-triggered average membrane potential time courses with large amplitude changes ranked according to trigger-preceding average membrane potential shown at the left of each trace. The number of family triggers indicated by the number in brackets to the right. Note that responses could be depolarizing, hyperpolarizing or a depolarization followed by a hyperpolarization. Right: summary plot of the total number of avalanche families and the number of families with select intracellular responses. Note that most families will not trigger a response in a randomly recorded pyramidal neuron. **(B)** Summary for ACSF condition in slices from young rats. **(C)** Summary for PTX condition.

We identified neurons with significant input during families by comparing membrane potential fluctuations before (−500 to −50 ms; baseline) and after (50–250 ms) family triggers. An SD in deflections at least three times bigger than the SD in baseline fluctuations (−500 to −50 ms) was considered significant. Out of 84 recorded cells, only about 50% received input from just a few avalanche families (for a summary see Figures 9A,B). Thus, we conclude that the participation of single neurons in avalanche dynamics was typically selective.

As in our *in vivo* results, the relationship between the input to the neuron and the population activity in Figures 7, Figures 8 would be missed or, at best, underestimated if one averaged over all avalanches and all neurons. Here we emphasize this point by computing the average membrane potential triggered on all avalanches except the selective family (Figures 7C,  8C; *control*), which results in no significant average membrane deflection. These results indicate that accounting for the spatial pattern of avalanches is crucial to identify the relationships we present. LFP activity recorded with a randomly chosen single electrode from our multi-site recordings is likely to be uncorrelated to the input to any, particularly patched neuron.

### Avalanche Diversity and Selectivity in Synaptic Input Is Abolished by Disinhibition

**Figure 10:**
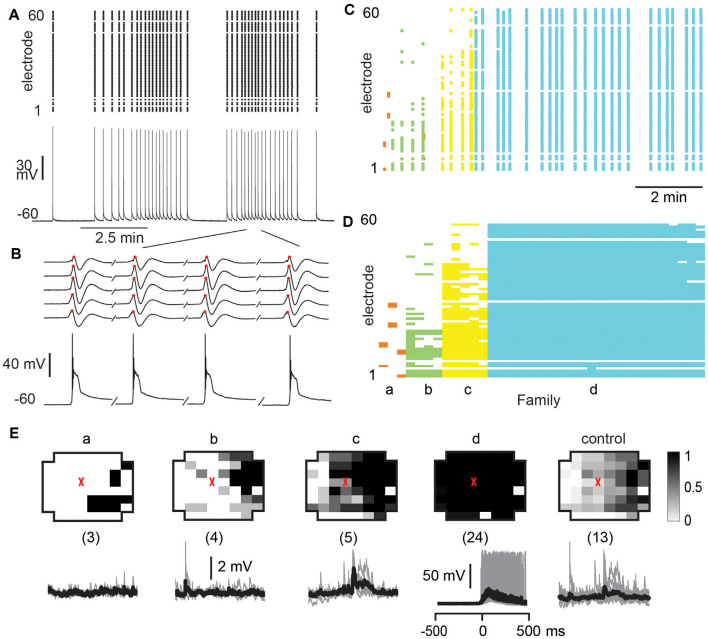
Global disinhibition reduces family diversity uncovering system-wide synchronization and tightly coupled neuronal firing. **(A)** Disinhibition induces synchronous nLFP activity on the MEA time-locked with a single neuron firing. PTX (50 μM) was bath applied at the beginning to block fast GABA_A_ receptor mediated inhibition. Top: nLFP raster. Bottom: intracellular membrane potential of a whole-cell patched pyramidal neuron. **(B)** Enlarged period in a demonstrating synchronous time course of nLFPs and corresponding large intracellular depolarization with spiking activity. Sequential occurrence of four population events grouped in time (discontinuity indicated by a forward slash). **(C)** nLFP raster on the MEA with color-coded avalanche families and their temporal occurrences. Note few families and tight firing for system-wide population event, identified as family *d*. **(D)** Corresponding family-sorted raster. **(E)** Most population events belong to family *d*, which spans most recording sites and always caused the patched cell to fire, i.e., the patched neuron was unselectively involved in network dynamics. Top: average patterns for each family. The red triangle marks the soma location of the patched neuron. The numbers of trigger events are shown in parentheses. Bottom: family-triggered average membrane potential waveforms (*black*). Pre-averaged individual traces are shown in gray. Vertical scale bars—2 mV (families *a*, *b*, and *c*), 50 mV (family *d*).

Finally, we investigated whether the selectivity encountered in our analysis might be due to a lack of excitability in the acute slice or might be maintained dynamically by the cortical network. To this end, we examined the role of fast GABA_A_-receptor mediated synaptic inhibition. It is well established that suppression of inhibition destroys avalanche dynamics *in vitro* (Beggs and Plenz, 2003; Stewart and Plenz, 2006; Pasquale et al., 2008). Accordingly, we tested whether the diversity and selectivity for families of ongoing LFP patterns depend on inhibitory signaling. We added the GABA_A_ receptor antagonist picrotoxin (50 μM) to our avalanche induction protocol for adult and young slices respectively. Under such disinhibited conditions, spontaneous nLFP count and rate increased by a factor of 10 in adult slices and by 50–100% in young slices (Table 1). Ongoing activity was comprised of stereotyped population events with large LFP amplitude and spatial extent resulting in bimodal size distribution of population events (Figure 6C, arrow; Figure 10). About three times fewer families were observed and a single slice-spanning family dominated most of the activity when compared to intact inhibition (Table 1). On average, neurons exhibited a 10 times loss in selectivity, i.e., most neurons participated in about half of all families and neurons revealed membrane depolarization or action potentials during nearly every population event (Figures 9C, 10; Table 1).

## Discussion

We simultaneously recorded single neuron and multi-site LFP activity from the cortex of awake monkeys and rat acute slices. In both preparations, spatiotemporal LFP patterns were distributed in sizes according to a power law, the hallmark of neuronal avalanches. The power-law quantifies a high incidence of large avalanches suggestive of a non-selective relationship between spatially extended LFP population signals and single-neuron activity. On the contrary, though, we found that diverse ensembles of extracellular units were selectively and reliably activated with particular avalanche patterns during ongoing activity in the premotor cortex of awake monkeys. We confirmed this selectivity in acute slices of rat cortex under two different activity levels, demonstrating reliable input to layer 2/3 pyramidal neurons during select and repeated LFP avalanches. We demonstrated that this selectivity breaks down during disinhibition and is not predicted by the spatially wide-spread correlation found with traditional spike-triggered or LFP-triggered average relationships. The selective participation of single neurons in repeated avalanches supports the view that avalanches are composed of highly diverse, yet selective neuronal ensembles.

Our work is related to previous studies in which multi-site recordings of LFP activity was compared to the activity of single-units (Destexhe et al., 1999; Rasch et al., 2008; Katzner et al., 2009; Nauhaus et al., 2009; Petermann et al., 2009; Kelly et al., 2010). Nauhaus et al. (2009) reported that the spiking activity of neurons generates negative LFP deflections near the neuron and decays with distance from the neuron. This conclusion was based on the spike-triggered average LFP recorded from anesthetized cats and monkeys. Our unit-triggered averages of LFP (Figure 1D) confirm these findings in awake monkeys. However, when lower frequency signals are not filtered out, i.e., 1–100 Hz is considered rather than 3–100 Hz as Nauhaus et al. (2009) did, the decay of the spike-triggered average LFP peak with distance is less prominent (Figure 1C). The converse relationship, i.e., LFP-triggered average spike histograms, revealed spatially widespread spiking during negative LFP deflections (Figure 1E). This observation is also consistent with previous observations of nLFP-triggered spike histograms (Destexhe et al., 1999; Petermann et al., 2009). Katzner et al. (2009) found that LFP signals originate from neurons within a 250 μm radius of the recording site. They reached this conclusion by comparing the orientation tuning of units and LFP signals in the visual cortex of anesthetized cats. Our results do not contradict this study, but we emphasize that neuronal avalanche dynamics is sensitive to anesthetics (Scott et al., 2014; Bellay et al., 2015) limiting extrapolations of previous findings to the current study in awake nonhuman primates. Indeed, previous studies have shown that LFP signals can be highly correlated over many millimeters of the cortex (Destexhe et al., 1999; Leopold and Logothetis, 2003; Nauhaus et al., 2009). When considered as a whole, our study demonstrates that large repeated population events involve selective ensembles of units distributed all across the 4 × 4 mm sized recording region. Some previous studies investigated the spike-LFP relationship by using spike trains to predict the LFP traces (Rasch et al., 2008) or vice versa (Kelly et al., 2010). Our work suggests that the success of such predictions would be substantially improved if algorithms take into account unit activity far from the LFP recording site as well the multi-site spatial pattern of the LFP.

Our analysis of population events are also related to previous studies using voltage-sensitive dye imaging (Tsodyks et al., 1999; Kenet et al., 2003; Han et al., 2008), which provides a spatially extended view of population activity similar to multi-site LFP recordings. As in our study, Kenet et al. (2003) and Han et al. (2008) found that population activity patterns repeat during ongoing cortical activity. Similar to our finding, Tsodyks et al. (1999) showed that a single neuron may fire selectively during certain ongoing “preferred cortical states,” which were defined by the spike-triggered average population pattern. However, our results indicate that single neurons are often selective for multiple different population events, specifically avalanche families, not just one “preferred cortical state.” Moreover, Tsodyks et al. (1999) restricted their attention to population events that resemble those caused by sensory stimuli, which, unlike our study, excludes the possibility that a neuron might be selected for an internal cognitive process unrelated to sensory stimulation.

The present study exclusively analyzed periods of “ongoing” activity, during which the animals did not perform any specific task but remained seated, with their head fixed. We considered ongoing activity *in vivo* to be the most appropriate comparison with spontaneous activity induced in our *in vitro* experiments. In the absence of a behavioral read-out during these periods, the origins of fluctuations in nLFP rate during ongoing activity are not known but might include visual saccades, spontaneous posture adjustments, minor limb movements, and changes in arousal amongst others. In a recent study from our group (Yu et al., 2017), we demonstrated scale-free LFP-avalanches during a simple movement task and a visual-motor mapping task in nonhuman primates suggesting that spontaneous, non-monitored movements should not qualitatively change our results.

Our objectives and the results of the *k*-means sorting were: (1) to establish several avalanche families, within which events had similar spatial patterns of activation; and (2) to identify a comparable number of families with relatively large spatial extent for both *in vivo* and *in vitro* data to facilitate comparison between the two approaches. Typically the number of *in vitro* LFP avalanches was at the order of ~10 lower than *in vivo*. In our view, there is no single “correct” choice of *k* for experimentally recorded cortical population events that are not likely to ever repeat exactly.

### The Complementary *In vivo* and *In vitro* Approaches in Assessing Single-Neuron Selectivity During Avalanches

Here we measured the relationship between LFP-based avalanches and single-neuron activity using two very different approaches, each of which provided distinct advantages as well as profound limitations. The advantages of our *in vivo* recordings are: (1) the embedding of MEA tips in an intact three-dimensional cortical space biased towards superficial layers in an awake animal; (2) that single-neuron activity was measured near the electrode tip ensuring a close spatial relationship between maximal local LFP activity and single-neuron activity; and (3) the ability to simultaneously probe many neurons from the same network. Major disadvantages are: (1) the limitation to neuronal firing that is single neuron *output*; and (2) the ambiguity as to LFP contributions from remote, potentially extracortical sources. Our *in vivo* analysis demonstrates that single neuron output is selective for avalanche families.

The advantages of our *in vitro* recordings are: (1) intracellular whole-cell patch recordings that allow for studying the neuronal *input* during avalanching; and (2) that LFP avalanches must arise from sources that are part of the local microcircuit. Major disadvantages of this approach are: (1) recording from only a few, typically 1 randomly selected neuron per slice; and (2) LFP recording sites being distant from the site of single-neuron recording and neuronal activity generation. The latter problem arises from the fact that in the acute slice, oxygen diffusion is limited to about 150 μm from the slice surface leading to a ~200 μm thick zone of hypoxic tissue separating the gas-impermeable planar MEA surface from the region of neuronal activity in the slice. With intracellular recording typically within the first 100 μm from the slice surface, we estimate that the typical distance between local LFP measurement sites and neuronal recording sites are of the order of 200–400 μm, much larger than *in vivo*. These spatial constraints should reduce the probability of finding tight coupling between the LFP and neuronal firing. This disadvantage as well as the low neuronal yield per experiment, though, is expected to be partially compensated for by dendritic arborizations of the recorded neurons which allow for subthreshold monitoring of remote network activity. Accordingly, our findings show that single neuron *input* is selective for avalanche families complementing our *in vivo* findings. Both approaches support the conclusion that the actual number of suprathreshold neurons during avalanching must be low even for large LFP patterns. Future experiments should target the selectivity encountered for single neurons during avalanches *in vivo*, for example using genetically encoded calcium indicators that monitor the neuronal firing, i.e., output, in combination with genetically encoded voltage indicators, which also monitor subthreshold activity (Knöpfel, 2012).

We note that when trying to assess the relationship between nLFP patterns and single-neuron activity, both extracellular and intracellular approaches naturally exhibit a bias against the identification of transient activity suppression. If extracellular unit firing is low, disfacilitation or active inhibition of firing is more difficult to identify because it can not be easily distinguished from unrelated quiet times in firing. For the intracellular membrane potential, active inhibition typically does not lead to a hyperpolarization unless the neuron is already depolarized. Both approaches thus bias detection of single-neuron—LFP relationships to transient excitation/depolarizations, potentially followed by suppression/hyperpolarization, in line with our experimental results.

### The Change in Neuronal Response and Avalanche Patterns Under Global Disinhibition *In vitro*

Bath-application of the GABA_A_-antagonist picrotoxin blocks inhibitory synaptic transmission in the cortical slice independent of the type of interneuron involved and subcellular location of the receptor. Non-selectively removing inhibition has been historically used *in vitro* (Beggs and Plenz, 2003; Pasquale et al., 2008) and in simulations (e.g., Tetzlaff et al., 2010) to collapse the power law in avalanche size into bimodal size distributions. Our study for the first time demonstrates this collapse to also drastically alter the relationship of single-neuron responses and spatially extended LFP patterns in the system. The non-selective reduction in GABA_A_-mediated, i.e., fast synaptic transmission, uncovered one to two orders of magnitude higher spontaneous activity levels compared to DA/NMDA induced avalanches in the adult slice (see Table 1). Specifically, the nLFP rate increased by a factor of 10, and total activity increased by a factor of 100 for similar recording periods (~20–25 min) besides expanding into deep layers (see Figure 6). This remarkable difference demonstrates that: (1) fast synaptic inhibition is required to induce and maintain avalanche dynamics; (2) avalanche dynamics allows layer-wide events to be produced despite the circuit being orders of magnitude below its full capacity in excitability; and (3) spontaneous avalanche activity is biased towards superficial layers despite deep layers in principle being excitable. Disinhibition in young slices also increased activity, yet less dramatically than in the adult slice confirming the general immature state of GABA_A_ mediated inhibition in the young cortex. These differences suggest developmental changes in the suppression of run-away excitation in the cortex and in the early support of avalanching in the neocortex.

Our family-triggered averages uncovered short-lasting depolarizations followed by hyperpolarization, or depolarizations and hyperpolarizations only. These subthreshold events and their respective order support the interpretation of inhibition being triggered by local recurrent excitation in the slice as an avalanche unfolds reminiscent of the synaptic “shadow” of a remotely propagating avalanche in the network.

To provide further insights into the inhibitory mechanisms involved in avalanche regulation, selective manipulation of distinct inhibitory microcircuit components e.g., interneuron cell types, will be required using e.g., optogenetical manipulation. Intracellular perfusion of whole-cell patched neurons with picrotoxin might allow for identifying excitatory inputs that underlie the selective sub- and suprathreshold responses of pyramidal neurons during avalanches.

### LFP Based Avalanches and Their Composition of Selective Neuronal Ensembles *In vivo*

Our treatment of LFP population events was motivated by our studies of neuronal avalanches identified in the LFP *in vitro* (Beggs and Plenz, 2003, 2004; Stewart and Plenz, 2006, 2007; Shew et al., 2009, 2011; Yang et al., 2012), *ex vivo* turtle cortex (Shew et al., 2015) and *in vivo* in the rat (Gireesh and Plenz, 2008) and nonhuman primate (Petermann et al., 2009; Yu et al., 2017; Miller et al., 2019). Our observations that spatial nLFP patterns repeat during ongoing activity was shown previously for neuronal avalanches, but only *in vitro* (Beggs and Plenz, 2004; Stewart and Plenz, 2006). A common view of LFP signals is that their physiological origins are too poorly understood to provide concrete information about cortical dynamics. Our work suggests that this view is due for an update. We show that traditional spike- and LFP-triggered average relationships are much weaker than the fluctuating moment-to-moment spike-LFP relationships. Individual units are not well represented by the “average unit” and individual LFP population events are not well represented by the “average event.” When these effects are accounted for, we show that diverse and reliable spiking ensembles underlie the cortical LFP-based avalanche.

Our work here demonstrates that neuronal avalanches are underpinned by selective, reliable spiking ensembles of neurons. This selectivity thus supports neuronal avalanches to be proposed (Plenz and Thiagarajan, 2007; Plenz, 2012) as a spatiotemporal organization of Hebbian cell assemblies (Hebb, 1949) lending strong experimental support to a large body of simulations on Hebbian plasticity, neuronal avalanches, and criticality (de Arcangelis et al., 2006; de Arcangelis and Herrmann, 2010; Rybarsch and Bornholdt, 2014; Stepp et al., 2015; Del Papa et al., 2017; Hernandez-Urbina and Herrmann, 2017; Michiels van Kessenich et al., 2018; Skilling et al., 2019; Zeng et al., 2019). By extension, the temporal organization of avalanches (Lombardi et al., 2014, 2016) or avalanches within avalanches (Petermann et al., 2009) and corresponding firing patterns of spike avalanches (Ribeiro et al., 2016) might provide a template for Hebb’s “phase sequences.”

## Data Availability Statement

The raw data supporting the conclusions of this article will be made available by the authors, without undue reservation.

## Ethics Statement

The animal study was reviewed and approved by the Animal Care and Use Committee of the National Institute of Mental Health, USA.

## Author Contributions

DP, SY, WS, and TB designed the research. SY, TB, WS, and JF-W performed the experiments. SY, TB, WS, and DP analyzed the data. TB, WS, SY, and DP discussed the results and wrote the manuscript. All authors contributed to the article and approved the submitted version.

## Conflict of Interest

The authors declare that the research was conducted in the absence of any commercial or financial relationships that could be construed as a potential conflict of interest.

## Footnotes

^1^https://imagej.net/Fiji

## References

[B1] Agrawal V. Cowley A. B. Alfaori Q. Larremore D. B. Restrepo J. G. Shew W. L. (2018). Robust entropy requires strong and balanced excitatory and inhibitory synapses. Chaos 28:103115. 10.1063/1.5043429 30384653

[B2] Beggs J. M. Plenz D. (2003). Neuronal avalanches in neocortical circuits. J. Neurosci. 23, 11167–11177. 10.1523/JNEUROSCI.23-35-11167.2003 14657176 PMC6741045

[B3] Beggs J. M. Plenz D. (2004). Neuronal avalanches are diverse and precise activity patterns that are stable for many hours in cortical slice cultures. J. Neurosci. 24, 5216–5229. 10.1523/JNEUROSCI.0540-04.2004 15175392 PMC6729198

[B4] Bellay T. Klaus A. Seshadri S. Plenz D. (2015). Irregular spiking of pyramidal neurons organizes as scale-invariant neuronal avalanches in the awake state. eLife 4:e07224. 10.7554/eLife.07224 26151674 PMC4492006

[B5] Bowen Z. Winkowski D. E. Seshadri S. Plenz D. Kanold P. O. (2019). Neuronal avalanches in input and associative layers of auditory cortex. Front. Syst. Neurosci. 13:45. 10.3389/fnsys.2019.00045 31551721 PMC6737089

[B6] Chialvo D. R. (2010). Emergent complex neural dynamics. Nat. Phys. 6, 744–750. 10.1038/nphys1803

[B7] Clawson W. P. Wright N. C. Wessel R. Shew W. L. (2017). Adaptation towards scale-free dynamics improves cortical stimulus discrimination at the cost of reduced detection. PLoS Comput. Biol. 13:e1005574. 10.1371/journal.pcbi.1005574 28557985 PMC5469508

[B8] de Arcangelis L. Herrmann H. J. (2010). Learning as a phenomenon occurring in a critical state. Proc. Natl. Acad. Sci. U S A 107, 3977–3981. 10.1073/pnas.0912289107 20160107 PMC2840167

[B9] de Arcangelis L. Perrone-Capano C. Herrmann H. J. (2006). Self-organized criticality model for brain plasticity. Phys. Rev. Lett. 96:028107. 10.1103/PhysRevLett.96.028107 16486652

[B10] Del Papa B. Priesemann V. Triesch J. (2017). Criticality meets learning: criticality signatures in a self-organizing recurrent neural network. PLoS One 12:e0178683. 10.1371/journal.pone.0178683 28552964 PMC5446191

[B11] Destexhe A. Contreras D. Steriade M. (1999). Spatiotemporal analysis of local field potentials and unit discharges in cat cerebral cortex during natural wake and sleep states. J. Neurosci. 19, 4595–4608. 10.1523/JNEUROSCI.19-11-04595.1999 10341257 PMC6782626

[B12] Ecker A. S. Berens P. Keliris G. A. Bethge M. Logothetis N. K. Tolias A. S. (2010). Decorrelated neuronal firing in cortical microcircuits. Science 327, 584–587. 10.1126/science.1179867 20110506

[B13] Fagerholm E. D. Scott G. Shew W. L. Song C. Leech R. Knöpfel T.. (2016). Cortical entropy, mutual information and scale-free dynamics in waking mice. Cerebral Cortex 26, 3945–3952. 10.1093/cercor/bhw200 27384059 PMC5028006

[B14] Gautam H. Hoang T. T. McClanahan K. Grady S. K. Shew W. L. (2015). Maximizing sensory dynamic range by tuning the cortical state to criticality. PLoS Comput. Biol. 11:e1004576. 10.1371/journal.pcbi.1004576 26623645 PMC4666488

[B15] Gireesh E. D. Plenz D. (2008). Neuronal avalanches organize as nested theta- and beta/gamma-oscillations during development of cortical layer 2/3. Proc. Natl. Acad. Sci. U S A 105, 7576–7581. 10.1073/pnas.0800537105 18499802 PMC2396689

[B16] Gollo L. L. (2017). Coexistence of critical sensitivity and subcritical specificity can yield optimal population coding. J. R. Soc. Interface 14:20170207. 10.1098/rsif.2017.0207 28954848 PMC5636266

[B17] Gray C. M. Singer W. (1989). Stimulus-specific neuronal oscillations in orientation columns of cat visual cortex. Proc. Natl. Acad. Sci. U S A 86, 1698–1702. 10.1073/pnas.86.5.1698 2922407 PMC286768

[B18] Han F. Caporale N. Dan Y. (2008). Reverberation of recent visual experience in spontaneous cortical waves. Neuron 60, 321–327. 10.1016/j.neuron.2008.08.026 18957223 PMC3576032

[B19] Hebb D. (1949). The Organization of Behavior. A Neuropsychological Theory. New York, NY: Wiley.

[B20] Hernandez-Urbina V. Herrmann J. M. (2017). Self-organized criticality *via* retro-synaptic signals. Front. Phys. 4:54 10.3389/fphy.2016.00054

[B21] Hesse J. Gross T. (2014). Self-organized criticality as a fundamental property of neural systems. Front. Syst. Neurosci. 8:166. 10.3389/fnsys.2014.00166 25294989 PMC4171833

[B22] Karimipanah Y. Ma Z. Miller J.-E. K. Yuste R. Wessel R. (2017). Neocortical activity is stimulus- and scale-invariant. PLoS One 12:e0177396. 10.1371/journal.pone.0177396 28489906 PMC5425225

[B23] Katzner S. Nauhaus I. Benucci A. Bonin V. Ringach D. L. Carandini M. (2009). Local origin of field potentials in visual cortex. Neuron 61, 35–41. 10.1016/j.neuron.2008.11.016 19146811 PMC2730490

[B24] Kelly R. C. Smith M. A. Kass R. E. Lee T. S. (2010). Local field potentials indicate network state and account for neuronal response variability. J. Comput. Neurosci. 29, 567–579. 10.1007/s10827-009-0208-9 20094906 PMC3604740

[B25] Kenet T. Bibitchkov D. Tsodyks M. Grinvald A. Arieli A. (2003). Spontaneously emerging cortical representations of visual attributes. Nature 425, 954–956. 10.1038/nature02078 14586468

[B26] Kinouchi O. Copelli M. (2006). Optimal dynamical range of excitable networks at criticality. Nat. Phys. 2, 348–351. 10.1038/nphys289

[B27] Klaus A. Yu S. Plenz D. (2011). Statistical analyses support power law distributions found in neuronal avalanches. PLoS One 6:e19779. 10.1371/journal.pone.0019779 21720544 PMC3102672

[B28] Knöpfel T. (2012). Genetically encoded optical indicators for the analysis of neuronal circuits. Nat. Rev. Neurosci. 13, 687–700. 10.1038/nrn3293 22931891

[B29] Leopold D. A. Logothetis N. K. (2003). Spatial patterns of spontaneous local field activity in the monkey visual cortex. Rev. Neurosci. 14, 195–205. 10.1515/revneuro.2003.14.1-2.195 12929926

[B30] Lombardi F. Herrmann H. Plenz D. de Arcangelis L. (2014). On the temporal organization of neuronal avalanches. Front. Syst. Neurosci. 8:204. 10.3389/fnsys.2014.00204 25389393 PMC4211381

[B31] Lombardi F. Herrmann H. J. Plenz D. de Arcangelis L. (2016). Temporal correlations in neuronal avalanche occurrence. Sci. Rep. 6:24690. 10.1038/srep24690 27094323 PMC4837393

[B32] Marković D. Gros C. (2014). Power laws and self-organized criticality in theory and nature. Phys. Rep. 536, 41–74. 10.1016/j.physrep.2013.11.002

[B33] Michiels van Kessenich L. Luković M. de Arcangelis L. Herrmann H. J. (2018). Critical neural networks with short- and long-term plasticity. Phys. Rev. E 97:032312. 10.1103/PhysRevE.97.032312 29776048

[B34] Miller S. R. Yu S. Plenz D. (2019). The scale-invariant, temporal profile of neuronal avalanches in relation to cortical γ-oscillations. Sci. Rep. 90:031001. 10.1038/s41598-019-52326-y 31712632 PMC6848117

[B35] Mora T. Bialek W. (2011). Are biological systems poised at criticality? J. Stat. Phys. 144, 268–302. 10.1007/s10955-011-0229-4

[B36] Muñoz M. A. (2017). Colloquium: criticality and dynamical scaling in living systems. Rev. Mod. Phys. 90:031001 10.1103/RevModPhys.90.031001

[B37] Murthy V. N. Fetz E. E. (1996). Synchronization of neurons during local field potential oscillations in sensorimotor cortex of awake monkeys. J. Neurophysiol. 76, 3968–3982. 10.1152/jn.1996.76.6.3968 8985893

[B38] Nauhaus I. Busse L. Carandini M. Ringach D. L. (2009). Stimulus contrast modulates functional connectivity in visual cortex. Nat. Neurosci. 12, 70–76. 10.1038/nn.2232 19029885 PMC2610236

[B39] Okun M. Naim A. Lampl I. (2010). The subthreshold relation between cortical local field potential and neuronal firing unveiled by intracellular recordings in awake rats. J. Neurosci. 30, 4440–4448. 10.1523/JNEUROSCI.5062-09.2010 20335480 PMC6634481

[B40] Pasquale V. Massobrio P. Bologna L. L. Chiappalone M. Martinoia S. (2008). Self-organization and neuronal avalanches in networks of dissociated cortical neurons. Neuroscience 153, 1354–1369. 10.1016/j.neuroscience.2008.03.050 18448256

[B41] Pesaran B. Pezaris J. S. Sahani M. Mitra P. P. Andersen R. A. (2002). Temporal structure in neuronal activity during working memory in macaque parietal cortex. Nat. Neurosci. 5, 805–811. 10.1038/nn890 12134152

[B42] Petermann T. Thiagarajan T. Lebedev M. A. Nicolelis M. A. Chialvo D. R. Plenz D. (2009). Spontaneous cortical activity in awake monkeys composed of neuronal avalanches. Proc. Natl. Acad. Sci. U S A 106, 15921–15926. 10.1073/pnas.0904089106 19717463 PMC2732708

[B43] Plenz D. (2012). Neuronal avalanches and coherence potentials. Eur. Phys. J. Spec. Top. 205, 259–301. 10.1140/epjst/e2012-01575-5

[B44] Plenz D. Thiagarajan T. C. (2007). The organizing principles of neuronal avalanches: cell assemblies in the cortex? Trends Neurosci. 30, 101–110. 10.1016/j.tins.2007.01.005 17275102

[B45] Rasch M. J. Gretton A. Murayama Y. Maass W. Logothetis N. K. (2008). Inferring spike trains from local field potentials. J. Neurophysiol. 99, 1461–1476. 10.1152/jn.00919.2007 18160425

[B46] Renart A. de la Rocha J. Bartho P. Hollender L. Parga N. Reyes A.. (2010). The asynchronous state in cortical circuits. Science 327, 587–590. 10.1126/science.1179850 20110507 PMC2861483

[B47] Ribeiro T. L. Ribeiro S. Copelli M. (2016). Repertoires of spike avalanches are modulated by behavior and novelty. Front. Neural Circuits 10:16. 10.3389/fncir.2016.00016 27047341 PMC4802163

[B48] Ribeiro T. L. Yu S. Martin D. A. Winkowski D. Kanold P. Chialvo D. R. (2020). Trial-by-trial variability in cortical responses exhibits scaling in spatial correlations predicted from critical dynamics. BioRxiv [Preprint]. 10.1101/2020.07.01.182014 PMC10956720 38341856

[B49] Rybarsch M. Bornholdt S. (2014). Avalanches in self-organized critical neural networks: a minimal model for the neural SOC universality class. PLoS One 9:e93090. 10.1371/journal.pone.0093090 24743324 PMC3990531

[B50] Sánchez J. Mardia K. Kent J. Bibby J. (1979). Multivariate Analysis. New York, NY: Academic Press.

[B51] Scott G. Fagerholm E. D. Mutoh H. Leech R. Sharp D. J. Shew W. L.. (2014). Voltage imaging of waking mouse cortex reveals emergence of critical neuronal dynamics. J. Neurosci. 34, 16611–16620. 10.1523/JNEUROSCI.3474-14.2014 25505314 PMC4261090

[B52] Shew W. L. Bellay T. Plenz D. (2010). Simultaneous multi-electrode array recording and two-photon calcium imaging of neural activity. J. Neurosci. Meth. 192, 75–82. 10.1016/j.jneumeth.2010.07.023 20659501 PMC2934901

[B53] Shew W. L. Clawson W. P. Pobst J. Karimipanah Y. Wright N. C. Wessel R. (2015). Adaptation to sensory input tunes visual cortex to criticality. Nat. Phys. 11, 659–663. 10.1038/nphys3370

[B54] Shew W. L. Yang H. Petermann T. Roy R. Plenz D. (2009). Neuronal avalanches imply maximum dynamic range in cortical networks at criticality. J. Neurosci. 29, 15595–15600. 10.1523/JNEUROSCI.3864-09.2009 20007483 PMC3862241

[B55] Shew W. L. Yang H. Yu S. Roy R. Plenz D. (2011). Information capacity is maximized in balanced cortical networks with neuronal avalanches. J. Neurosci. 5, 55–63. 10.1523/JNEUROSCI.4637-10.2011 21209189 PMC3082868

[B56] Shriki O. Yellin D. (2016). Optimal information representation and criticality in an adaptive sensory recurrent neuronal network. PLoS Comput. Biol. 12:e1004698. 10.1371/journal.pcbi.1004698 26882372 PMC4755578

[B57] Skilling Q. M. Ognjanovski N. Aton S. J. Zochowski M. (2019). Critical dynamics mediate learning of new distributed memory representations in neuronal networks. Entropy 21:1043 10.3390/e21111043

[B58] Stepp N. Plenz D. Srinivasa N. (2015). Synaptic plasticity enables adaptive self-tuning critical networks. PLoS Comput. Biol. 11:e1004043. 10.1371/journal.pcbi.1004043 25590427 PMC4295840

[B59] Stewart C. V. Plenz D. (2006). Inverted-U profile of dopamine-NMDA-mediated spontaneous avalanche recurrence in superficial layers of rat prefrontal cortex. J. Neurosci. 26, 8148–8159. 10.1523/JNEUROSCI.0723-06.2006 16885228 PMC6673780

[B60] Stewart C. V. Plenz D. (2007). Homeostasis of neuronal avalanches during postnatal cortex development *in vitro*. J. Neurosci. Meth. 169, 405–416. 10.1016/j.jneumeth.2007.10.021 18082894 PMC2743406

[B61] Tetzlaff C. Okujeni S. Egert U. Wörgötter F. Butz M. (2010). Self-organized criticality in developing neuronal networks. PLoS Comput. Biol. 6:e1001013. 10.1371/journal.pcbi.1001013 21152008 PMC2996321

[B62] Tsodyks M. Kenet T. Grinvald A. Arieli A. (1999). Linking spontaneous activity of single cortical neurons and the underlying functional architecture. Science 286, 1943–1946. 10.1126/science.286.5446.1943 10583955

[B63] Yang H. Shew W. L. Roy R. Plenz D. (2012). Maximal variability of phase synchrony in cortical networks with neuronal avalanches. J. Neurosci. 32, 1061–1072. 10.1523/JNEUROSCI.2771-11.2012 22262904 PMC3319677

[B64] Yu S. Klaus A. Yang H. Plenz D. (2014). Scale-invariant neuronal avalanche dynamics and the cut-off in size distributions. PLoS One 9:e99761. 10.1371/journal.pone.0099761 24927158 PMC4057403

[B65] Yu S. Ribeiro T. L. Meisel C. Chou S. Mitz A. Saunders R.. (2017). Maintained avalanche dynamics during task-induced changes of neuronal activity in nonhuman primates. eLife 6:e27119. 10.7554/eLife.27119 29115213 PMC5677367

[B66] Yu S. Yang H. Nakahara H. Santos G. S. Nikolic D. Plenz D. (2011). Higher-order interactions characterized in cortical activity. J. Neurosci. 31, 17514–17526. 10.1523/JNEUROSCI.3127-11.2011 22131413 PMC6623824

[B67] Zeng G. Huang X. Jiang T. Yu S. (2019). Short-term synaptic plasticity expands the operational range of long-term synaptic changes in neural networks. Neural Netw. 118, 140–147. 10.1016/j.neunet.2019.06.002 31254768

